# 艾迪联合紫杉醇和顺铂治疗晚期非小细胞肺癌的*meta*分析

**DOI:** 10.3779/j.issn.1009-3419.2010.11.06

**Published:** 2010-11-20

**Authors:** 权 王, 曦冉 何, 金徽 田, 小刚 汪, 沛帆 茹, 自良 阮, 克虎 杨

**Affiliations:** 1 730000 兰州，兰州大学循证医学中心，兰州大学基础医学院 Evidence Based Medicine Center of Lanzhou University, School of Basic Medical Science of Lanzhou University, Lanzhou 730000, China; 2 730000 兰州，兰州大学第一临床医学院 the First Clinical Medical College of Lanzhou University, Lanzhou 730000, China

**Keywords:** 艾迪注射液, 化疗, 肺肿瘤, *meta*分析, Aidi injection, Chemotherapy, Lung neoplasms, *meta* analysis

## Abstract

**背景与目的:**

艾迪联合紫杉醇和顺铂（paclitaxel and cisplatin, TP）治疗晚期非小细胞肺癌（non-small cell lung cancer, NSCLC）在临床疗效和安全性方面是否优于单用紫杉醇和顺铂存在着争议。本研究系统评价艾迪联合TP化疗方案治疗晚期NSCLC的临床疗效和安全性。

**方法:**

计算机检索Cochrane Library、Pubmed、EMBASE、CancerLit、中国生物医学文献数据库、中国期刊全文数据库、中文科技期刊全文数据库，检索时间从各数据库建库至2010年3月20日；同时辅助其它检索，纳入艾迪联合TP化疗方案治疗NSCLC的随机对照试验（randomized controlled trials, RCTs）。两名评价者独立评价纳入研究的质量并提取资料，并用RevMan 5.0软件进行统计分析。

**结果:**

共纳入11篇RCTs，*meta*分析结果显示：与单纯TP化疗方案相比，艾迪注射液联合TP化疗方案可以改善近期疗效（RR=1.27, 95%CI: 1.10-1.47, *P*=0.001）、提高生活质量（RR=1.85, 95%CI: 1.54-2.21, *P* < 0.001）、减少白细胞下降（RR=0.71, 95%CI: 0.57-0.87, *P*=0.001）和血小板下降（RR=0.59, 95%CI: 0.40-0.87, *P*=0.008），降低恶心呕吐等胃肠道反应（RR=0.75, 95%CI: 0.58-0.98, *P*=0.03），而在血红蛋白下降（RR=0.97, 95%CI: 0.70-1.34, *P*=0.85）、肝功能下降（RR=0.63, 95%CI: 0.09-1.57, *P*=0.18）、肾功能下降（RR=0.42, 95%CI: 0.14-1.24, *P*=0.12）、周围神经炎发生情况（RR=0.86, 95%CI: 0.56-1.32, *P*=0.50）和脱发（RR=0.92, 95%CI: 0.63-1.34, *P*=0.66）方面的差异无统计学意义。

**结论:**

艾迪注射液联合TP方案可提高NSCLC治疗的近期疗效和患者生活质量、改善骨髓抑制，并降低化疗所产生的不良反应，值得临床推广使用。

## Abstract (en)

**Background and objective:**

Compared with chemotherapy, whether aidi injection can improve the patient's quality of life is not definite. The aim of this study is to evaluate the efficacy and safety of axotere plus eisplatin chemotherapy combining aidi injection for patients with non-small cell lung cancer (NSCLC).

**Methods:**

We searched relevant randomized controlled trials (RCTs) from Cochrane library, Pubmed, EMBASE, CancerLit, VIP, CBM and CNKI *etc*. The search was finished in March 20, 2010. We traced the related references and experts in this field, besides we also communicated with other authors to obtain some certain information that has not been found. RCTs of aidi injection plus TP versus TP for advanced NSCLC were included. We evaluated the quality of these included studies and analyzed data by Cochrane Collaboration's RevMan 5.0 software.

**Results:**

Eleven RCTs involving 800 patients were included. *meta* analysis results suggested that compared with TP chemotherapy alone, the combination had a statistically significant benefit in healing efficacy (RR=1.2, 95%CI: 1.10-1.47, *P*=0.001) and improving quality of life (QOL) (RR=1.85, 95%Cl: 1.54-2.21, *P* < 0.001). Besides, the combination also had a statistically significant benefit in myelosuppression, white blood cell (WBC)(RR=0.71, 95%CI: 0.57-0.87, *P*=0.001) and hematoblast (RR=0.59, 95%CI: 0.40-0.87, *P*=0.008) and in reducing the gastroenteric reaction (RR=0.75, 95%CI: 0.58-0.98, *P*=0.03). But the combination had no statistically significant benefit in prevention of reducing hemoglobin (Hb) (RR=0.97, 95%CI: 0.70-1.34, *P*=0.85), liver function (RR=0.63, 95%CI: 0.09-1.57, *P*=0.18), kidney function (RR=0.42, 95%CI: 0.14-1.24, *P*=0.12), peripheral neuritis (RR=0.86, 95%CI: 0.56-1.32, *P*=0.50), and baldness (RR=0.92, 95%CI: 0.63-1.34, *P*=0.66).

**Conclusion:**

Compared with TP chemotherapy alone, the combination can significantly improve the efficiency, QOL and myelosuppression, and reduce adverse events.

肺癌是严重危害人类健康的疾病，根据世界卫生组织（World Health Organization, WHO）2003年公布的资料显示，肺癌无论是发病率（120万/年）还是死亡率（110万/年）均居全球癌症的首位。在我国，肺癌超过了癌症死因的20%，且发病率和死亡率均迅速增长。自2000年至2005年，我国肺癌的发病人数增加了11.6万，死亡人数增加了10.1万^[1]^。据WHO预测，到2025年，我国每年新增的肺癌病死例数将超过100万，患者数将居世界之首^[2]^。其中，非小细胞肺癌（non-small cell lung cancer, NSCLC）约占肺癌的80%-85%，而其中1/3患者初次确诊时已为局部晚期，失去了手术机会，其5年生存率不足5%^[3]^。

化疗是中晚期肺癌综合治疗的主要手段之一，多西他赛和顺铂（paclitaxel and cisplatin, TP）组成的TP方案对晚期NSCLC的疗效肯定，有效率高，能延长患者的生存期，并能使晚期NSCLC患者的中位生存期延长1个月-3个月，1年生存率增加10%^[4]^。TP方案治疗晚期NSCLC的有效率为25%-53%^[5, 6]^。TP化疗方案作为治疗NSCLC的最有效的方案之一，其化疗毒副反应大，导致患者生活质量下降，增加患者痛苦^[7]^。因此，迫切需要新的辅助治疗方法，以改善症状、提高生存率、减少副作用。近年来，中药配合化疗治疗恶性肿瘤的研究进展提示，中药与化疗同用可降低各种毒副作用，改善患者生活质量，提高化疗的疗效^[8]^。

艾迪注射液是临床广泛应用的一种抗肿瘤中药，根据中医学中扶正祛邪的原理，采用黄芪、刺五加、人参与去甲斑蝥素精制而成，主要含有人参皂苷、黄芪皂苷、刺五加多糖和去甲斑蝥素等，具有扶正、祛邪、清热、解毒的功效，既有调节增强免疫功能又有抗肿瘤作用^[9]^。

为了客观评价艾迪注射液联合TP方案治疗晚期NSCLC的疗效，本研究收集所有关于艾迪注射液联合TP方案治疗晚期NSCLC的随机对照试验（randomized controlled trials, RCTs），采用Cochrane系统评价方法，客观评价艾迪注射液联合TP方案治疗晚期NSCLC的有效性和安全性，以期为临床医生合理用药提供真实可靠的依据。

## 1 材料与方法

### 1.1 纳入排除标准

#### 1.1.1 研究设计

RCT，无论是否采用盲法。

#### 1.1.2 研究对象

纳入标准：①18岁以上经病理/细胞学检查证实的晚期NSCLC的患者，性别、种族及国籍等均不限；②卡氏评分≥60分或ECOG评分为0分-2分；③之前未接受化学治疗；④治疗前无化疗禁忌症，肝肾功能、血液学、心电图无明显异常者。排除标准：①伴有严重内科疾患及感染；②同时伴随其它恶性肿瘤；③肺癌为其它肿瘤转移病灶。

#### 1.1.3 干预措施

艾迪注射液联合TP方案*vs*单用TP方案。

#### 1.1.4 测量指标

有效性指标：有效率、生活质量；安全性指标：毒副反应，主要包括血液学毒性和胃肠道反应以及其它毒性反应等。

### 1.2 检索策略

计算机检索Cochrane Library、Pubmed、EMBASE、CancerLit、中国生物医学文献数据库、中国期刊全文数据库、中文科技期刊全文数据库，检索时间从各数据库建库至2010年3月20日。检索词包括：non-small cell lung cancer、non small cell lung carcinoma、non small cell lung carcinomas、non small cell lung、NSCLC、ai di、aidi、ai-di、非小细胞肺癌、艾迪注射液、艾迪注射剂、爱迪注射液和爱迪注射剂等。RCT检索策略遵循Cochrane系统评价手册5.0.2，其它检索采用主题词与自由词相结合的方式，并根据具体数据库调整，所有检索策略通过多次预检索后确定。另外，补充检索了中国RCT数据库，并用Google Scholar、Medical martix等搜索引擎在互联网上查找相关的文献，追查已纳入文献的参考文献，与本领域的专家、通讯作者等联系，以获取以上检索未发现的相关信息。

### 1.3 文献筛选和资料提取

两位研究者交叉核对纳入研究的结果，对有分歧而难以确定其是否纳入的研究通过讨论或第3位研究者决定是否纳入。缺乏的资料通过电话或信件与作者联系予以补充。提取数据主要内容包括：①一般资料：题目、作者姓名、发表日期和文献来源；②研究特征：研究对象的一般情况、各组患者的基线可比性及干预措施；③结局指标。如遇分歧通过讨论或根据第3位研究人员的意见协商解决。如试验报告不详或资料缺乏，通过信件与作者进行联系获取。

### 1.4 质量评价

根据Cochrane系统评价手册推荐的质量评价方法使用统一的质量评价表对纳入研究进行方法学质量评价：①采用何种随机分配方法，方法是否正确；②是否进行分配隐藏，方法是否正确；③是否采用盲法，对哪些人实施了盲法；④有无失访或退出。如果所有4条质量评价标准均完全满足，则该研究存在偏倚的可能性最小；如果其中任何一条或多条质量评价标准仅为部分满足或不清楚，则该研究存在中度偏倚的可能性；如果其中任何一条或多条质量评价标准完全不满足，则该研究存在高度偏倚的可能性。质量评价由2位研究者独立进行并交叉核对，如遇分歧通过讨论或请第3位研究者协助解决。

### 1.5 统计学处理

采用Cochrane协作网提供的RevMan 5.0统计软件^[10]^进行*meta*分析。计数资料采用相对危险度（relative risk, RR）为疗效分析统计量，计量资料采用均数差（mean difference, MD），各效应量均以95%CI表示。各纳入研究结果间的异质性采用*χ*^2^检验，若*P*>0.1和*I*^2^ < 50%，采用固定效应模型进行分析，若存在统计学异质性（*P* < 0.1, *I*^2^50%）时，分析异质性来源，确定是否能采用随机效应模型。如果研究间存在明显的临床异质性，只对其进行描述性分析。必要时，采用敏感性分析检验结果的稳定性。

## 2 结果

### 2.1 纳入研究概述（图 1）

按照检索策略和资料收集方法，共查到相关文献249篇，通过阅读标题、摘要进行初筛后纳入文献117篇；利用Endnote X2软件去重以及进一步阅读文献排除重复或不符合纳入标准的文献100篇，可能符合标准的文献有17篇，再经过阅读全文按纳入标准及数据完整性进行筛选，共纳入11项RCT^[11-21]^，共800例患者。

**1:**
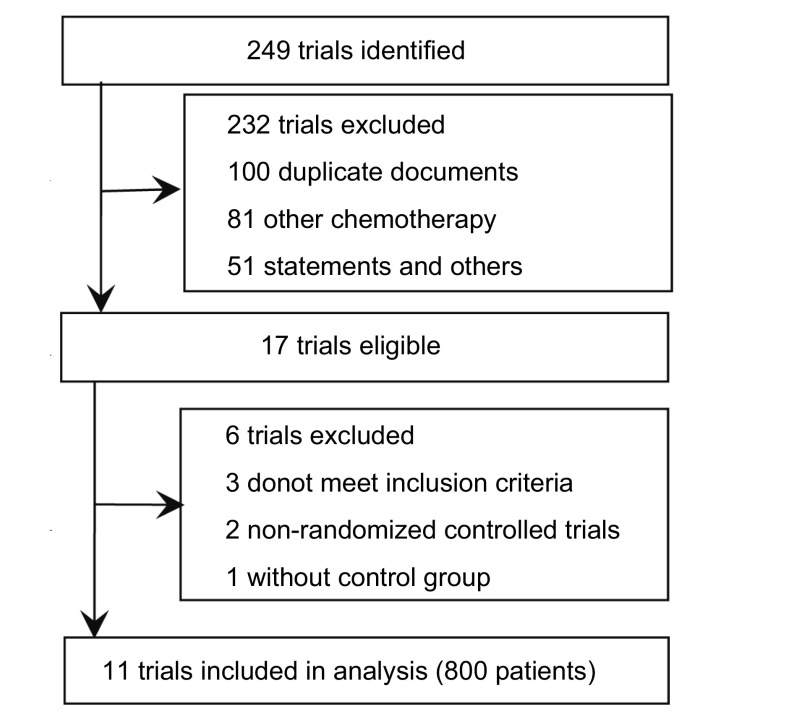
纳入研究流程图 Selection of trials

### 2.2 纳入研究一般情况（表 1）

**1:** 纳入研究的一般情况 The characteristics of included studies

Included studies	Cases			Age (Years) (average^*^)			Sex (M/F)		Aidi injection (mL)	Time (day)	Outcome
	Aidi+TP	TP		Aidi+TP	TP		Aidi+TP	TP			
Bian GM^[11]^	34	30		(62.3^*^)	(61.8^*^)		23/11	21/9	50	56	①②③④⑤⑥⑦	
Chen XM^[12]^	32	32		47-72 (64^*^)	49-71 (62^*^)		21/11	18/14	50	42	①②③④
Hou EC^[13]^	35	35		34-69 (52^*^)	37-70 (51^*^)		22/13	19/16	50	42	①②③④⑧
Li XD^[14]^	48	48		—	—		—	—	50	56	①②③
Lin Q^[15]^	30	30		35-72 (55^*^)	37-73 (57^*^)		21/9	20/10	50	28	①②③④
Peng RT^[16]^	32	32		42-75 (54.6^*^)	45-72 (53.7^*^)		22/10	20/12	50	42	①②③
Su W^[17]^	32	32		33-75 (55^*^)	34-75 (54^*^)		20/12	19/13	50	42	①②③④
Wang HM^[18]^	40	40		—	—		—	—	80-100	≥42	①②③④⑤
Yang G^[19]^	40	42		28-75	30-77		28/12	28/14	50-100	42	①②③④⑤⑥
Zhou H^[20]^	50	46		36-63 (56^*^)	35-65 (54^*^)		32/18	28/18	50	42	①③④⑤⑥⑦
Zhu QS^[21]^	30	30		36-74 (57^*^)	33-74 (59^*^)		15/15	17/13	50	63	①②③
①efficacy rate; ②quality of life; ③myelosuppression; ④gastrointestinal reaction; ⑤damage of liver and kidney; ⑥neurotoxicity; ⑦alopecia; ⑧immune function.											

纳入研究均无中医病、证的诊断及描述，10项研究^[11-20]^采用了WHO实体瘤评价标准^[22]^，1项研究^[21]^采用了《实体瘤疗效评价标准》（Response Evaluation Criteria in Solid Tumors, RECIST）^[23]^评价；10项研究^[11-20]^以Karnofsky评分评价生活质量，1项研究^[21]^分级不一致未纳入合并分析。在治疗作用方面，11项研究^[11-21]^均报道了近期瘤体大小改变的有效率。2项研究^[12, 15]^报道了临床受益率。1项研究^[17]^报道了临床症状。1项研究^[12]^报道了中位生存率。在骨髓抑制方面，11项研究均采用WHO毒性反应标准^[22]^。11项研究^[11-21]^均报道了白细胞下降情况。6项研究^[14, 15, 18-21]^报道了血小板下降情况。3项研究^[14, 17, 20]^报道了对血红蛋白变化的影响。在胃肠道反应方面，8项研究^[11-13, 15, 17-20]^报告了恶心呕吐为主的消化道症状，1项研究^[21]^报道了腹泻。在脏器毒性方面，4项研究^[11, 18-20]^报道了肝脏毒性和肾脏毒性，1项研究^[13]^报道了心脏毒性。在其它方面的研究中，3项研究^[11, 19, 20]^报道了神经毒性，2项研究^[11, 20]^报道了对脱发的影响，1项研究^[19]^报道了肌肉疼痛，1项研究^[13]^报道了免疫功能的改变。

### 2.3 纳入研究质量评价（表 2）

**2:** 纳入研究方法学质量评价 Quality assessment of methodology of included studies

Included studies	Sequence generation	Allocated concealment	Blinding	Withdraw	Fellow-up	Quality grading
Bian GM^[11]^	Unclear	Unclear	Unclear	No	Unclear	B
Chen XM^[12]^	Unclear	Unclear	Unclear	No	Unclear	C
Hou EC^[13]^	Envelope	Unclear	Unclear	No	Unclear	B
Li XD^[14]^	Unclear	Unclear	Unclear	No	Unclear	B
Lin Q^[15]^	Unclear	Unclear	Unclear	No	Unclear	C
Peng RT^[16]^	Unclear	Unclear	Unclear	No	Unclear	B
Su W^[17]^	Unclear	Unclear	Unclear	No	Unclear	B
Wang HM^[18]^	Unclear	Unclear	Unclear	No	Unclear	B
Yang G^[19]^	Unclear	Unclear	Unclear	No	Unclear	B
Zhou H^[20]^	Unclear	Unclear	Unclear	No	Unclear	C
Zhu QS^[21]^	Random digits table	Unclear	Unclear	No	Unclear	B

纳入研究均提及随机分组，1项研究^[21]^采用随机数字表进行随机分组，1项研究^[13]^采用纸袋法进行随机分组。对其余研究经电话联系作者核实文献方法学质量，3项研究^[12, 15, 20]^按患者入院顺序分配，为半RCT，其余研究不清楚；纳入研究均未描述是否采用盲法和分配隐藏，均无退出和失访，纳入研究基线均可比。

### 2.4 *meta*分析结果

#### 2.4.1 有效率（图 2）

**2:**
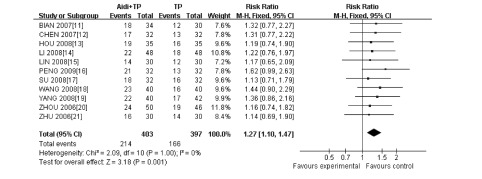
瘤体变化有效率的*meta*分析 *meta* -analysis of the included trials on effective rate

11项研究^[11-21]^报告了有效率，共有800例患者，其中艾迪联合TP方案组406例患者，TP方案组394例患者，各研究间无异质性（*P*=1.00, *I*^2^=0%），采用固定效应模型。*meta*分析结果显示，两组在有效率方面差异有统计学意义（RR=1.27, 95%CI: 1.10-1.47, *P*=0.001）。

#### 2.4.2 生活质量的改善（图 3）

**3:**
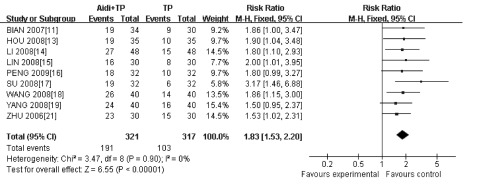
生存质量的*meta*分析 *meta* -analysis of the included trials on quality of life

与单用TP方案比较，艾迪注射液联合TP方案能更好地改善患者生活质量，二者比较差异有统计学意义（RR=1.83, 95%CI: 1.53-2.20, *P* < 0.001）。

#### 2.4.3 毒副反应的影响（表 3）

**3:** 毒副反应的*meta*分析 *meta*-analysis of the included trials on adverse effects

Outcome	Number of included studies	Aidi injection plus TP			TP			Heterogeneity		Analysis model	Statistical method	Effect estimate	
		Events	Total		Events	Total		*P*	*I*^2^			RR (95%CI)	*P*
Hemotologic toxicity											*Mantel-Haenazel*		
White blood cell	11 ^[11-21]^	200	403		275	397		< 0.001	78%	Random effect		0.71 [0.57, 0.87]	0.001
Platelet	6^[14, 17-21]^	32	240		52	238		0.55	0%	Fixed effect		0.59 [0.40, 0.87]	0.008
Hemoglobin	3 ^[14, 17, 20]^	57	130		57	126		0.28	22%			0.94 [0.76, 1.18]	0.61
Gastroenteric reaction													
Nausea/vomiting	8^[11-13, 15, 17-20]^	123	291		164	289		0.02	57%	Random effect	*Mantel-Haenazel*	0.75 [0.58, 0.98]	0.03
Alopecia	2^[11, 20]^	26	84		26	76		0.46	0%	Fixed effect	*Mantel-Haenazel*	0.92 [0.63, 1.34]	0.66
Other toxicity										Fixed effect	*Mantel-Haenazel*		
Liver	4^[11, 18-20]^	13	164		20	158		0.58	0%			0.63 [0.32, 1.23]	0.18
Kidney	4^[11, 18-20]^	4	164		10	158		0.99	0%			0.42 [0.12, 1.24]	0.12
Heart	1^[13]^	3	35		9	35		-	-			0.33 [0.10, 1.13]	0.08
Nerve	3^[11, 19, 20]^	29	124		32	116		0.46	0%			0.86 [0.56, 1.32]	0.50

##### 2.4.3.1 血液学毒性

与对照组比较，艾迪注射液联合TP方案对造血功能具有保护作用。其结果显示：在减少白细胞下降方面，二者比较差异有统计学意义（RR=0.71, 95%CI: 0.57-0.87, *P*=0.001）；在减少血小板下降方面二者比较差异亦有统计学意义（RR=0.59, 95%CI: 0.40-0.87, *P*=0.008）；但在减少血红蛋白方面的差异无统计学意义（RR=0.94, 95%CI: 0.76-1.18, *P*=0.61）。

##### 2.4.3.2 消化道反应

与对照组比较，艾迪注射液联合TP方案对恶心呕吐具有保护作用。其结果显示：在减少恶心呕吐的发生率方面，二者比较差异有统计学意义（RR=0.75, 95%CI: 0.58-0.98, *P*=0.03）。1项研究^[21]^报道了腹泻情况结果显示：两组≥Ⅲ度腹泻情况比较无统计学差异（*P*>0.05）。

##### 2.4.3.3 脱发

艾迪注射液联合TP方案在减少脱发方面与单纯TP方案比较，差异无统计学意义（RR=0.92, 95%CI: 0.63-1.34, *P*=0.66）。

##### 2.4.3.4 其它毒性

艾迪注射液联合TP方案对肝、肾以及神经的保护作用与单纯TP方案比较，两组差异均不具有统计学意义（表 3）。且1项研究^[13]^报道了心脏毒性结果，显示两组比较差异无统计学意义（*χ*^2^=3.62, *P*>0.05）。

#### 2.4.4 发表性偏倚（图 4）

对纳入文献进行了漏斗图分析。结果显示漏斗图图形不对称，提示可能存在发表性偏倚。

**4:**
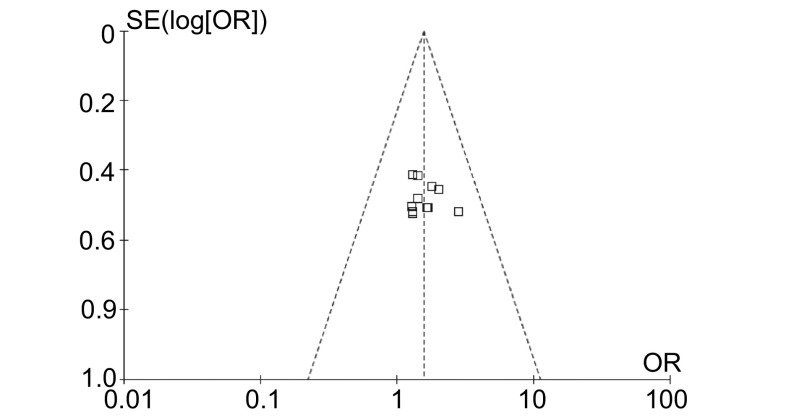
漏斗图 Funnel plot

## 3 讨论

艾迪注射液是从人参、黄芪、刺五加、斑蝥等中草药中经现代工艺提取而成的中药制剂，具有抗肿瘤及免疫调节等多靶点治疗功能。研究^[24]^报道，化疗联合艾迪注射液治疗晚期NSCLC可以减轻化疗不良反应，提高生活质量。

本研究结果显示，与单用TP方案相比，艾迪注射液联合TP方案可以提高有效率、改善生活质量、降低患者化疗后血小板减少和白细胞减少的发生率，同时减少胃肠道反应等。但由于检索文献时，并未限定语种、年限和样本量及存在发表偏倚，本研究通过阅读题名及摘要，排除非随机、回顾性对照研究，进一步阅读全文，剔除不符合纳入标准的文献，并对纳入文献的参考文献进行扩大检索，以提高研究的科学性。

各纳入研究间病例纳入排除标准相似，同时各研究都对治疗前年龄、性别、治疗情况等因素进行了基线一致性分析，单用TP方案组和艾迪注射液联合TP方案组具有可比性。本研究纳入的11篇文献方法学质量相对较低，除2篇外^[5, 8]^无一篇文献描述随机方法，9篇文献仅提及随机字样，无一篇文献正确实施分配隐藏和盲法。但由于肿瘤化疗的特殊性，往往难以采用随机方案的隐藏和盲法，故虽然纳入的RCT具有发生偏倚的高度可能性，但对于化疗药物治疗肿瘤的临床研究而言，仍可认为是较高质量的RCT。纳入11项RCT的结果，均提示艾迪注射液TP方案对NSCLC化疗可能有一定辅助治疗作用。

本系统评价纳入研究存在以下局限性，从而会影响结果的论证强度：①大多数研究均未提及样本量估算的依据，很多研究样本量都较小，这将导致检验效能低；②所纳入的研究在药物的使用量及使用时间上不一致，这对最终的测量指标将会产生一定的影响；③本系统评价纳入研究中无一项描述是否实施了隐蔽分组。据调查，不实施或不充分实施隐蔽分组会夸大疗效；④由于在评价疗效时部分采用主观性指标，故盲法的使用在研究艾迪注射液联合TP方案治疗NSCLC的研究中很重要，人为因素影响结果的可能性较大，而未充分实施盲法会直接导致实施偏倚和测量偏倚的发生；⑤纳入研究对部分结果采用不同的统计量，致使有些试验不能合并，无法得出统一结论。但总体而言，本系统评价纳入研究质量尚可，对结果有一定的论证强度，但在上述几点尚需改进。

综上所述，艾迪注射液联合TP化疗方案在治疗NSCLC方面，优于单纯使用TP化疗方案。合并分析显示艾迪注射液对TP化疗方案的NSCLC患者有一定的辅助治疗作用。但本系统评价所纳入的研究质量不高，临床研究方法不完全正确，且参加研究的病例数较少，上述结论存在偏倚的可能较大，所以尚需设计严密、实施科学的大样本研究进一步证实。

## References

[1] Lu ZY, Zhong NS (2009). Internal Medicine.

[2] Bai CX, Zhang X (2006). Status quo for lung cancer treatment. Chin J Tubercul Resp.

[3] Rivera MP (2004). Multimodality therapy in the treatment of lung cancer. Semin Respir Crit Care Med.

[4] Schiller JH, Harrington D (2002). Comparison of four chemotherapy regimens for advanced non small cell lung cancer. N Engl J Med.

[5] Millward MJ, Zalcberg J, Bishop JF (1997). Phase Ⅰ trial of docetaxel and cisplatin in previously untreated patients with advanced non small cell lung cancer. J Clin Oncol.

[6] Chevalier T, Monnier A, Douillard JY (1998). Docetaxel (Taxoòtere) plus cisplatin: an active and well tolerated combination in patients with advanced non small cell lung cancer. Eur J Cancer.

[7] Zhu GY, Li SH, Zhang SF (2010). The clinical progress of aidi injection. Acta Chin Med Pharmacol.

[8] Wang DN (2008). Advanced research on the induced-toxicity of chemotherapy by Chinese traditional medicine. Lishizhen Med Medica Res.

[9] Li HJ, Dong L, Li Y (2007). The effect of aidi injection for T lymphocyte subsets of patients with advanced tumors. J Tradit Chinese Med.

[b10] 10Review Manager (RevMan)[Computer program]. Version 5. 0. Copenhagen: The Nordic Cochrane Cencer, The Cochrane Collaboration, 2008.

[11] Bian MG, Xing HY, Luo Y (2007). Clinical observation of taxotere plus cisplatin chemotherapy in combination with aidi injection in the treatment of nonsmall cell advanced lung cancer. Liaoning J Trad Chinese Med.

[12] Chen XM, Gong HT, Song ZJ (2007). Randomized study of aidi injection plus TP regimen combination in the treatment of advanced non-small cell lung cancer. Chin J Cancer Prev Treat.

[13] Hou EC (2008). A clinical study on combined with TP regimen in treatment of patients with advanced non-small cell lung cancer. World Health Digest.

[14] Li XD, Wang WF (2008). Clinical observation of aidi injection combined with chemotherapy for treatment of the advanced non-small cell lung cancer. Chin J Misdiag.

[15] Lin Q (2008). Clinical observation of taxotere plus cisplatin chemotherapy in combination with aidi injection in the treatment of 60 cases of non-small cell advanced lung cancer. Zhejiang Clin Med J.

[16] Peng RT (2009). Treatment of 32 cases of advanced non-small cell lung cancer with aidi injection combined with taxotere plus cisplatin chemotherapy. Jiangxi J Trad Chinese Med.

[17] Su W, Wang Y, Li HT (2008). Clinical observation of taxotere plus cisplatin chemotherapy in combination with aidi injection in the treatment of non-small cell advanced lung cancer. Chin J Misdiag.

[18] Wang HM, Liao GQ, Liu PH (2008). Aidi injection combined with chemotherapy for treatment of the advanced non-small cell lung cancer. Chin J Clin Oncol Rehabilit.

[19] Yang G, Jiang HX, Wang B (2008). Treatment of 40 cases of late non-small cell lung cancer with aidi injection combined with chemotherapies. Clin Focus.

[20] Zhou H, Wang AL, Yi Q (2006). A clinical study on paclitaxel and cisplation chemotherapy combined with aidi injection in treatment of advanced nonsmall cell lung cancer. Tumor J World.

[21] Zhu QS, Chen YM (2006). Clinical observation of taxotere plus cisplatin chemotherapy in combination with aidi injection in the treatment of non-small cell advanced lung cancer. Chin J Pharmacoepiolemiol.

[22] Sun Y (2001). Medical Oncology.

[23] Tsuchida Y, Therasse P (2001). Response evaluation criteria in solid tumors (RECIST): New guidelines. Med Pediatr Oncol.

[24] Wang DJ, Chen YL, Ren J (2004). A randomized clinical study on efficacy of aidi injection combined with chemotherapy in the treatment of advanced non-small cell lung cancer. Chin J Lung Cancer.

